# Clinical features and risk factors of postoperative in-hospital mortality following surgical repair of Stanford type A acute aortic dissection

**DOI:** 10.1186/s12872-021-02107-1

**Published:** 2021-08-12

**Authors:** Chen Ke, Hao Wu, Min Xi, Wei Shi, Qihong Huang, Guirong Lu

**Affiliations:** 1 Department of Cardiothoracic Surgery, The Affiliated Hospital of Hangzhou Normal University, 126 Wenzhou Road, Hangzhou, 310029 China; 2 General Ward of Internal Medicine, Dingqiao Hospital, Hangzhou, China

**Keywords:** Acute aortic dissection, Clinical features, Death, Risk factor, Surgery

## Abstract

**Background:**

To investigate the clinical features of patients with Stanford type A acute aortic dissection (AAD) and analyze the risk factors affecting postoperative in-hospital mortality rate.

**Methods:**

The demographic and clinical data were retrospectively collected and analyzed from 118 AAD patients admitted to the Affiliated Hospital of Hangzhou Normal University from June 2016 to April 2019. All patients underwent surgical treatment and were grouped into death and survival groups. The risk factors affecting postoperative in-hospital death were analyzed using multivariate logistic regression analysis.

**Results:**

The male to female ratio in the patients was 3.8:1 and the mean age was 50.11 ± 9.91 years. The patient’s main comorbidities were hypertension (70.33%) and coronary heart disease (10.17%). The main symptoms included chest pain and back pain (72.89%). The highest incidence of complications was pericardial effusion (48.31%), followed by pleural effusion (22.88%). The mean systolic blood pressure, white blood cell count and D-dimer in the patients were over the ranges of normal people. The incidences of cardiac and renal insufficiency were 18.64% and 16.95% respectively, and the postoperative in-hospital mortality rate was 12.71%. Univariable analysis showed that age, renal insufficiency, cardiac insufficiency, D-dimer level, cardiopulmonary bypass time, operation time, blood transfusion volume and postoperative hemostasis were significant factors leading to the death (*P* < 0.05). Multivariate logistic regression analysis showed that age > 65, renal insufficiency, cardiopulmonary bypass time ≥ 250 min and postoperative hemostasis were independent risk factors for the death (*P* < 0.05).

**Conclusions:**

AAD patients frequently have underlying diseases with pain as the main symptom. Age > 65 years, renal insufficiency, cardiopulmonary bypass time ≥ 250 min and postoperative hemostasis are significantly risk factors for postoperative mortality.

## Background

Acute aortic dissection (AAD) is a common severe cardiovascular disease with high mortality [1, 2]. Based on a multi-institutional retrospective study, the emergency department incidences of AAD vary between 5.93/100,000 and 24.92/100,000 [3]. As a life-threatening condition, early diagnosis, treatment and close follow-up are critical to improve the survival of patients. The main pathology in AAD results from cystic degenerative lesions in the middle layer of the aorta. The lesion could result in a local tear of the intima, leading to the formation of hematoma that may expand longitudinally along the aorta due to the strong impact and infiltration of blood flow [4]. AAD patients sometimes present predominantly with neurological symptoms from cerebral ischemia [4] and often have sudden and severe pain in the chest, back and abdomen [5]. 60–70% of AAD patients are affected at the ascending aorta, which are classified as Stanford type A [6]. Nontraumatic Stanford type A acute aortic dissection (NTAD) is a life-threatening condition. Without intervention, the mortalities at 48 h and 2 weeks from onset are 50 and 80%, respectively [7]. This dissection in Stanford type A AAD often involves the ascending aorta proximal to the brachiocephalic trunks, the aortic arch and the descending aorta. Because the tear may occur in different sites and multiple organs are involved in this type of patients, Stanford type A AAD is more complicated than Stanford type B AAD. It often occurs acutely with rapid progress and poor prognosis, seriously threatening the life of patients [8, 9].

Surgical treatments are increasingly recognized as an important option to improve the survival rate and life quality of AAD patients due to technology advance in brain protection, anesthesia and postoperative monitoring [10, 11]. A number of surgical procedures have been developed to deal with various clinical conditions with minimal cerebral and cardiac ischemia in the past 20 years [12–14]. However, despite of considerable improvement, postoperative mortality is still an issue for AAD, which could be as high as 27.4% [15–17]. The patients who undergo surgical operations often have severe postoperative complications [18, 19] and require reoperation [20]. Therefore, further improvement is needed for surgical treatments to achieve better prognosis and outcomes, particularly less mortality. Therefore, identification of risk factors for surgical treatment would help improve the outcomes. Earlier study showed that age, cerebral ischemia, acute renal injury and spinal cord injury are among the important risk factors that affect postoperative mortality and complications [17, 21, 22].

In this study, we analyzed the clinical characteristics of AAD patients to identify additional factors contributing to in-hospital mortality after surgical repair of Stanford type A AAD. The findings would help improve the surgical outcomes and prognosis of AAD patients.

## Subjects and methods

### Subjects and surgical treatment

AAD patients admitted to the Affiliated Hospital of Hangzhou Normal University, Hangzhou, between June 2016 and April 2019 were retrospectively reviewed. Patients were included if he/she was radiologically proven to have AAD according to the American Heart Association criteria [23], underwent Sun’s procedure for AAD repair and had complete set of clinical data with a disease course of < 2 weeks. Patients were excluded if he/she had traumatic AAD, pseudoaneurysm, Marfan syndrome, Ehlers–Danlos syndrome, vasculitis and connective tissue disease. Patients with congenital aortic malformation and intramural hematoma were also excluded. Patients were also excluded if they had other malignant diseases and immunodeficiency diseases, such as cancers. All patients received Sun’s procedure for AAD repair as described earlier [24] by the same team of eight physicians. This study was approved by the Ethic Committee of Hangzhou Normal University and written informed consent was obtained from every participant.

### Data collection

Basic data, clinical data and surgical data were collected from the hospital medical databases. Basic data included demographic data, such as age, gender, education level, medical history and underlying diseases such as hypertension, diabetes and coronary heart disease. Clinical data included clinical manifestations such as symptoms and complications (pericardial effusion, aortic regurgitation, lower extremity ischemia, nervous system symptoms, hypotension or shock, pleural effusion), laboratory examination findings such as blood pressure (systolic pressure, diastolic pressure), leukocyte count, D-dimer level, cardiac and renal dysfunctions such as cardiac insufficiency (patients with heart failure and having symptoms such as difficulty breathing with physical activity, the inability to lie flat and chest tightness) and renal insufficiency (patients with poor renal functions who may have multiple manifestations such as fluid overload, hyperkalemia, metabolic acidosis, abnormalities of calcium, phosphorus, and vitamin D metabolism, and anemia). Operation-related data such as cardiopulmonary bypass time, cerebral ischemia time, operation time, blood transfusion volume, and postoperative thoracotomy for hemostasis were also collected. The patients were divided into death group if died in the hospital before discharge or survival groups if lived at discharge.

### Statistical analysis

Statistical software (SPSS22.0) was used for statistical analysis. The normality of distribution of continuous variables was tested by one-sample Kolmogorov–Smirnov test. Measurement data with normal distribution were expressed as mean ± SD (standard derivation) and were compared using the Student’s t-test. Counting data were expressed as percentage and were compared using *X*^2^ test or the Fisher exact probability test. Multivariate logistic regression analysis was performed to identify the risk factors related to postoperative death. A value of *P* < 0.05 was considered statistically significant.

## Results

### Demographic and clinical features of AAD patients

A total of 118 patients were included in the study and their demographic and clinical data are presented in Table 1. There were 83 males and  35 females. They were aged between 23.5 and 77.2 years with a mean age of 50.11 ± 9.91 years. The comorbidities included hypertension (70.33% (83/118)), coronary heart disease (10.17% (12/118)), diabetes (15.25% (18/118)) and others. Patients were admitted with pain and distress presented in the chest (34.75% (41/118)), back (38.14% (45/118)) and abdomen (20.34% (24/118)). They had complications such as pericardial effusion (48.31% (57/118), aortic regurgitation (22.88% (27/418)), pleural effusion (21.19% (25/118)) and lower extremity ischemia (10.17% (12/118))). A few of them had neurological symptoms (2.54% (3/118)), hypotension (1.69% (2/118)) and shock (1.69% (2/118)). Laboratory findings showed that the mean systolic blood pressure, mean diastolic blood pressure and white blood cell counts were 121.24 mmHg, 79.32 mmHg and 7.89 ± 0.99 × 10^3^ cells/mL. Mean D-dimer level was 2.50 ± 0.55 mg/L. Cardiac insufficiency and renal insufficiency were observed in 18.64% (22/118) and 16.95% (20/118) of the patients. Compared with patients in the death group, patients in the survival group had significantly lower age, more female, more hypertension, lower DDi level, less aortic regurgitation, fewer cardiac insufficiency and fewer renal insufficiency (*P* < 0.05, Table 1).

**Table 1:** Demographic characteristics and clinical data of patients

Variables	All patients (n =118)	Death group (n = 15)	Survival group (n = 103)	*t/X*^*2*^	*P* value
Age (year)	50.11 ± 9.91	68.14 ± 8.96	40.21 ± 3.91	9.541	0.001
Sex, female (%)	26.31	18.52	27.47	2.766	0.024
BMI (kg/m^2^)	25.80 ± 3.44	24.30 ± 3.14	25.60 ± 2.15	0.445	0.124
Hypertension (%)	66.51	70.37	65.92	3.444	0.012
Coronary heart disease (%)	11.32	11.11	11.35	0.151	0.812
Diabetes mellitus (%)	15.35	20.35	13.65	1.453	0.135
Cerebral stroke (%)	2.50	2.77	2.40	0.898	0.135
Chronic obstructive pulmonary disease (%)	2.11	2.01	2.13	0.443	0.235
Smoking (%)	12.51	13.51	12.11	0.737	0.123
Alcohol use (%)	6.23	6.13	6.53	0.228	0.223
White blood cell (×10^3^/mL)	7.89 ± 0.99	6.89 ± 0.87	7.99 ± 0.69	0.567	0.723
Hemoglobin (g/dL)	15.06 ± 0.92	14.76 ± 0.76	15.86 ± 0.79	0.212	0.263
Creatinine (µmol/L)	72.00 ± 3.90	79.10 ± 2.11	71.99 ± 3.91	1.332	0.073
Albumin (mg/dL)	0.87 ± 0.21	0.72 ± 0.28	0.96 ± 0.25	1.654	0.223
DDi (mg/L)	2.50 ± 0.55	4.10 ± 0.85	1.50 ± 0.25	6.014	0.001
Estimated glomerular filtration rate (mL/min/1.73 m²)	62.11 ± 5.44	60.11 ± 3.14	62.71 ± 6.74	0.867	0.153
Systolic blood pressure (mmHg)	121.24 ± 11.55	122.14 ± 10.45	120.94 ± 12.15	0.922	0.213
Diastolic blood pressure (mmHg)	79.32 ± 8.98	77.12 ± 8.18	80.12 ± 9.18	1.922	0.113
AAD pain site					
Chest pain (%)	34.89	32.19	34.99	0.342	0.213
Back pain (%)	38.00	37.10	38.55	0.182	0.643
Abnormal pain (%)	20.12	25.12	19.99	0.543	0.642
Others (%)	6.99	5.99	7.15	0.182	0.392
Complications					
Pericardial effusion (%)	43.81	38.89	44.54	0.282	0.231
Aortic regurgitation (%)	18.90	31.48	17.07	11.282	0.000
Pleural effusion (%)	21.05	20.02	21.55	0.482	0.078
Cardiac insufficiency (%)	22.56	33.30	20.98	8.221	0.001
Renal insufficiency (%)	15.00	27.78	13.10	6.22	0.023

### Univariable analysis of postoperative in-hospital death

Among the included patients, 15 died before discharge from the hospital, accounting for 12.71% of all patients. Univariable analysis showed that there were significant differences in age, hypertension, renal insufficiency, cardiac insufficiency, D-dimer level, cardiopulmonary bypass time, operation time, blood transfusion volume and postoperative hemostasis between the two groups (*P* < 0.05, Table 2). On other hand, no difference was observed in gender, and comorbidities, such as hypertension, coronary heart disease, type II diabetes and complications such as aortic regurgitation and cerebral ischemia (*P* < 0.05, Table 2).

**Table 2:** Univariable analysis of factors affecting postoperative in-hospital mortality in AAD patients

Variable	Death group		Survival group		*X*^2^	*P*
	N	%	%	%		
Age (years)					10.303	0.001
> 65	36	66.67	41	41.05		
≤ 65	18	33.33	215	58.95		
Sex					1.939	0.164
Male	44	81.48	264	72.49		
Female	10	18.52	100	27.51		
Comorbidity with hypertension					0.305	0.581
Yes	38	70.37	240	65.94		
No	16	29.63	124	34.06		
Comorbidity with coronary heart disease					0.003	0.995
Yes	6	11.11	41	11.35		
No	48	88.89	323	88.65		
Comorbidity with diabetes					0.876	0.321
Yes	23	42.59	177	48.63		
No	31	57.41	187	51.37		
Renal insufficiency					6.859	0.009
Yes	15	27.78	48	17.86		
No	39	72.22	316	82.14		
Cardiac insufficiency					3.905	0.048
Yes	18	33.33	76	20.96		
No	36	66.67	288	79.04		
Pericardial effusion					0.715	0.398
Yes	21	38.89	162	44.54		
No	33	61.11	202	55.46		
Aortic regurgitation					2.177	0.140
Yes	17	31.48	62	17.03		
No	37	68.52	302	82.97		
DDi level (mg/L)					6.103	0.013
≥ 5.00	31	57.41	143	39.30		
< 5.00	23	42.59	221	60.70		
Cardiopulmonary bypass time (min)					37.015	0.000
≥ 250 min	44	81.48	127	34.93		
< 250 min	10	18.52	237	65.07		
Cerebral ischemia (m)					1.851	0.174
≥ 30.00	22	40.74	159	43.67		
< 30.00	32	59.26	205	56.33		
Operation time (h)					3.957	0.047
≥ 7.50	30	55.56	148	40.61		
< 7.50	24	44.44	216	59.39		
Blood transfusion (L)					9.040	0.003
≥ 3.00	29	53.70	168	46.29		
< 3.00	25	46.30	196	53.71		
Postoperative thoracotomy for hemostasis					11.140	0.001
Yes	14	25.93	29	7.86		
No	40	74.07	335	92.14		

### Multivariate logistic regression analysis of postoperative in-hospital death

The results of logistic regression analysis showed that the independent risk factors of in-hospital death in the AAD patients were age > 65 years old, renal insufficiency, cardiopulmonary bypass time ≥ 250 min and postoperative hemostasis (*P* < 0.05, Table 3). The odd ratios (OR) of these parameters ranged from 1.009 in age and 6.938 in cardiopulmonary bypass time.

**Table 3:** Significant risk factors of postoperative in-hospital mortality identified by multivariate logistic regression analysis in patients with AAD

Variable	B	SE	Wald *X*^2^	*P*	OR	95% CI
Age > 65years	0.023	0.099	4.212	0.040	1.009	1.005–1.421
Cardiac insufficiency	0.876	0.232	2.123	0.082	2.114	0.652–7.314
Renal insufficiency	1.121	0.343	6.335	0.011	5.273	2.158–19.439
D-dimer ≥ 5.00 mg/L	1.331	0.334	2.210	0.119	2.156	0.869–9.312
Cardiopulmonary bypass time ≥ 250 min	0.872	0.224	5.876	0.010	6.938	2.231–18.810
Operative time ≥ 7.50 h	2.221	0.876	1.223	0.183	2.254	0.815–8.122
Blood transfusion volume ≥ 3.0 L	1.321	0.123	2.186	0.097	2.117	0.814–8.216
Postoperative hemostasis	3.121	0.667	5.271	0.025	3.807	1.128–10.935

## Discussion

Significant advances in the diagnosis and therapy have greatly improved surgical outcomes of acute type A dissection [16]. However, with the increase of the incidence rate of various cardiovascular diseases, such as hypertension and coronary heart disease, the incidence of AAD is increasing year by year, and it tends to occur younger. The reported postoperative mortality rate for AAD patients is between 5 and 27.4% [15–17]. In this cohort, the mortality rate is 12.92%, indicating that the surgical procedures still have certain risk for AAD patients and further efforts are needed to improve the prognosis and outcomes. Earlier studies showed that age, preoperative stroke, preoperative shock and cardiopulmonary bypass time are risk factors of death in AAD patients, but the operation methods, aortic intubation and perfusion are not related to postoperative death [21].

Since surgical treatments of AAD are complicated, the outcomes are influenced by many preoperative, intraoperative and postoperative conditions. A better understanding of the clinical characteristics of AAD patients and analysis of the risk factors of postoperative death are of great significance for improving the prognosis and treatment planning.

This study found that the ratio of male to female in this AAD cohort is 3.8:1, which is slightly lower than the ratio reported previously [25]. This is likely due to sampling variation. The mean age is 50.11 ± 9.91 years, which is slightly younger than the age reported in earlier studies [26], suggesting that the patients trend to be younger. The etiology and pathogenesis of AAD have not been fully elucidated [27] and are generally believed to be associated with hypertension, coronary heart disease and other factors [28]. This study showed that the AAD patients have higher comorbidities with hypertension (70.33%) and coronary heart disease (10.17%) than previous report [24]. These differences might be attributed to dietary habits and living styles in different regions, as well as to the awareness and detection rate of chronic diseases in the current population. Hypertension and coronary heart disease are shown to be associated with the incidence of AAD [17]. AAD patients in this study were mainly admitted to the hospital with pain at different sites, which is consistent with other studies [29, 30]. AAD causes occlusion, ischemia, hematoma and other changes in the blood circulation that lead to complications such as pericardial effusion, aortic regurgitation, pleural effusion and lower extremity ischemia. In this cohort, pericardial effusion and aortic regurgitation are the most common, which is consistent with the previous studies [31]. Systolic blood pressure, leukocyte count and D-dimer level are also used as auxiliary indicators for AAD [32]. Our study showed that the AAD patients have elevated mean systolic pressure, leukocyte count and D-dimer level. These results are slightly different from the previous results [33, 34]. The increased mean systolic pressure may be related to decompensation for myocardial ischemia in the patients, while the increased leukocyte count might result from inflammation due to the tear of vascular intima. D-dimer level is an important functional indicator for the myocardium. Higher D-dimer level indicates reduced myocardial function [35] and could be observed in patients with AAD [32].

This study also found that some AAD patients had renal insufficiency and cardiac insufficiency, suggesting that for these patients the dissection have implicated renal artery, leading to poor bilateral perfusion. The cardiac insufficiency might be due to the involvement of dissection at the coronary ostium, causing hemodynamic changes and affecting cardiac function as reported previously [36].

To investigate the factors that might impact the post-operative in-hospital mortality rate, logistic regression analysis was performed. The results showed that age > 65 years, renal insufficiency, cardiopulmonary bypass time ≥ 250 min and postoperative hemostasis are independent risk factors for postoperative death (*P* < 005). It is likely that older patients would have more underlying diseases and weaken function of organs such as heart, lung and kidney [37] and thus more vulnerable to surgical injury. Renal insufficiency is associated with insufficient perfusion in renal artery, affecting hemodynamics, water and electrolyte metabolism and acid-base balance. After surgical trauma, patients with renal insufficiency would have particularly negative impact in patients undergoing vascular surgery and endovascular therapy [38]. Long cardiopulmonary bypass would activate inflammatory reaction, destroy the coagulation mechanism and cause serious damage to important organs [39]. Hemostasis following surgery often needs thoracotomy that will generate more trauma to patients, and excessive bleeding that requires hemostasis might also be an indicator of poor coagulation mechanism for patients and increase the risk of death.

There are a number of limitations in the study. It was a single center, retrospective study with relatively small sample. The patients were from local areas and not followed up for long term. Some of important clinical data such as structural or tissue disease, and ECG were not included due to data availability. Furthermore, medication information was not collected analyzed. Therefore, multiple-center, large and prospective studies with more parameters, particularly more laboratory tests, are needed to validate our conclusions.

## Conclusions

AAD patients trend to be younger in recent years and many have underlying diseases such as hypertension. Age > 65 years, renal insufficiency, cardiopulmonary bypass time ≥ 250 min and postoperative hemostasis are significantly risk factors for postoperative mortality.

## Data Availability

The datasets used and/or analysed during the current study are available from the corresponding author on reasonable request.

## Abbreviations

AAD: Acute aortic dissection
NTAD: Nontraumatic Stanford type A acute aortic dissection
SD: Standard derivation

## References

[1] Yoshida K, Nagasawa A, Koyama T (2018). Successful repair of acute aortic dissection after stent placement into the occluded left common carotid artery; report of a case. Kyobu Geka.

[2] Gaul C, Dietrich W, Friedrich I, Sirch J, Erbguth FJ (2007). Neurological symptoms in type A aortic dissections. Stroke.

[3] Wundram M, Falk V, Eulert-Grehn JJ, Herbst H, Thurau J, Leidel BA, Goncz E, Bauer W, Habazettl H, Kurz SD (2020). Incidence of acute type A aortic dissection in emergency departments. Sci Rep.

[4] Ohara T, Koga M, Tokuda N, Tanaka E, Yokoyama H, Minatoya K, Nagatsuka K, Toyoda K, Minematsu K (2016). Rapid identification of type A aortic dissection as a cause of acute ischemic stroke. J Stroke Cerebrovasc Dis.

[5] Pisano C, Rita Balistreri C, Fabio Triolo O, Argano V, Ruvolo G (2016). Acute type A aortic dissection: beyond the diameter. J Heart Valve Dis.

[6] Funakoshi H, Mizobe M, Homma Y, Nakashima Y, Takahashi J, Shiga T (2018). The diagnostic accuracy of the mediastinal width on supine anteroposterior chest radiographs with nontraumatic Stanford type A acute aortic dissection. J Gen Fam Med.

[7] Coady MA, Rizzo JA, Goldstein LJ, Elefteriades JA (1999). Natural history, pathogenesis, and etiology of thoracic aortic aneurysms and dissections. Cardiol Clin.

[8] Zhao L, Chai Y, Li Z (2017). Clinical features and prognosis of patients with acute aortic dissection in China. J Int Med Res.

[9] Karube N, Imoto K (2015). Emergency surgical treatment of Stanford type A acute aortic dissection. Kyobu Geka.

[10] Liu N, Zhang W, Ma W, Shang W, Zheng J, Sun L (2017). Risk factors for hypoxemia following surgical repair of acute type A aortic dissection. Interact Cardiovasc Thorac Surg.

[11] Kato M, Ohnishi K, Kaneko M, Ueda T, Kishi D, Mizushima T, Matsuda H (1996). New graft-implanting method for thoracic aortic aneurysm or dissection with a stented graft. Circulation.

[12] Karck M, Chavan A, Hagl C, Friedrich H, Galanski M, Haverich A (2003). The frozen elephant trunk technique: a new treatment for thoracic aortic aneurysms. J Thorac Cardiovasc Surg.

[13] Sun L, Li M, Zhu J, Liu Y, Chang Q, Zheng J, Qi R (2011). Surgery for patients with Marfan syndrome with type A dissection involving the aortic arch using total arch replacement combined with stented elephant trunk implantation: the acute versus the chronic. J Thorac Cardiovasc Surg.

[14] Brueck M, Heidt MC, Szente-Varga M, Bandorski D, Kramer W, Vogt PR (2006). Hybrid treatment for complex aortic problems combining surgery and stenting in the integrated operating theater. J Interv Cardiol.

[15] Ma WG, Zhu JM, Zheng J, Liu YM, Ziganshin BA, Elefteriades JA, Sun LZ (2013). Sun’s procedure for complex aortic arch repair: total arch replacement using a tetrafurcate graft with stented elephant trunk implantation. Ann Cardiothorac Surg.

[16] Uchida K, Karube N, Yasuda S, Miyamoto T, Matsuki Y, Isoda S, Goda M, Suzuki S, Masuda M, Imoto K (2016). Pathophysiology and surgical treatment of type A acute aortic dissection. Ann Vasc Dis.

[17] Pansini S, Gagliardotto PV, Pompei E, Parisi F, Bardi G, Castenetto E, Orzan F, di Summa M (1998). Early and late risk factors in surgical treatment of acute type A aortic dissection. Ann Thorac Surg.

[18] Amano J, Kuwano H, Yokomise H (2013). Thoracic and cardiovascular surgery in Japan during 2011: annual report by The Japanese Association for Thoracic Surgery. Gen Thorac Cardiovasc Surg..

[19] Easo J, Weigang E, Holzl PP, Horst M, Hoffmann I, Blettner M, Dapunt OE, group Gs (2012). Influence of operative strategy for the aortic arch in DeBakey type I aortic dissection: analysis of the German Registry for Acute Aortic Dissection Type A. J Thorac Cardiovasc Surg.

[20] Bekkers JA, Raap GB, Takkenberg JJ, Bogers AJ (2013). Acute type A aortic dissection: long-term results and reoperations. Eur J Cardiothorac Surg.

[21] Chu J, Wei J, Chang Q, Zhang H (2017). Risk factors of mortality and morbidity after surgical procedure for Stanford type A aortic dissection. Chin J Clin Thorac Cardiovasc Surg.

[22] Trimarchi S, Eagle KA, Nienaber CA, Rampoldi V, Jonker FH, De Vincentiis C, Frigiola A, Menicanti L, Tsai T, Froehlich J (2010). Role of age in acute type A aortic dissection outcome: report from the International Registry of Acute Aortic Dissection (IRAD). J Thorac Cardiovasc Surg.

[23] O’Gara PT, Kushner FG, Ascheim DD, Casey DE, Chung MK, de Lemos JA, Ettinger SM, Fang JC, Fesmire FM, Franklin BA (2013). 2013 ACCF/AHA guideline for the management of ST-elevation myocardial infarction: executive summary: a report of the American College of Cardiology Foundation/American Heart Association Task Force on Practice Guidelines: developed in collaboration with the American College of Emergency Physicians and Society for Cardiovascular Angiography and Interventions. Catheter Cardiovasc Interv.

[24] Sun L, Qi R, Zhu J, Liu Y, Chang Q, Zheng J (2011). Repair of acute type A dissection: our experiences and results. Ann Thorac Surg.

[25] Jiang DS, Yi X, Zhu XH, Wei X (2016). Experimental in vivo and ex vivo models for the study of human aortic dissection: promises and challenges. Am J Transl Res.

[26] Xia L, Li J, Zhao K (2015). Incidence and in-hospital mortality of acute aortic dissection in China: analysis of China Health Insurance Research (CHIRA) Data 2011. J Geriatr Cardiol.

[27] Fann JI, Sarris GE, Mitchell RS, Shumway NE, Stinson EB, Oyer PE, Miller DC (1990). Treatment of patients with aortic dissection presenting with peripheral vascular complications. Ann Surg.

[28] Merkle J, Sabashnikov A, Deppe AC, Zeriouh M, Eghbalzadeh K, Weber C, Rahmanian P, Kuhn E, Madershahian N, Kroener A (2018). Impact of hypertension on early outcomes and long-term survival of patients undergoing aortic repair with Stanford A dissection. Perfusion.

[29] Koga M, Iguchi Y, Ohara T, Tahara Y, Fukuda T, Noguchi T, Matsuda H, Minatoya K, Nagatsuka K, Toyoda K (2018). Acute ischemic stroke as a complication of Stanford type A acute aortic dissection: a review and proposed clinical recommendations for urgent diagnosis. Gen Thorac Cardiovasc Surg.

[30] Conzelmann LO, Weigang E, Mehlhorn U, Abugameh A, Hoffmann I, Blettner M, Etz CD, Czerny M, Vahl CF, Investigators G (2016). Mortality in patients with acute aortic dissection type A: analysis of pre- and intraoperative risk factors from the German Registry for Acute Aortic Dissection Type A (GERAADA). Eur J Cardiothorac Surg.

[31] Thunberg CA, Ramakrishna H (2015). Echocardiographic detection of intimo-intimal intussusception in a patient with acute Stanford type A aortic dissection. Ann Card Anaesth.

[32] Zhang D, Shen C (2019). The correlation between levels of serum D-dimmer and MMP-9 and acute lung injury in acute type A aortic dissection. Label Immunoass Clin Med..

[33] Zhou X, Wang R, Zhang T, Liu F, Zhang W, Wang G, Gu G, Han Q, Xu D, Yao C (2019). Identification of lysophosphatidylcholines and sphingolipids as potential biomarkers for acute aortic dissection via serum metabolomics. Eur J Vasc Endovasc Surg.

[34] Goda M, Minami T, Imoto K, Uchida K, Masuda M, Meuris B (2018). Differences of patients’ characteristics in acute type A aortic dissection - surgical data from Belgian and Japanese centers. J Cardiothorac Surg.

[35] Aydin S, Ugur K, Aydin S, Sahin I, Yardim M (2019). Biomarkers in acute myocardial infarction: current perspectives. Vasc Health Risk Manag.

[36] Pan X, Lu J, Cheng W, Yang Y, Zhu J, Jin M (2018). Independent factors related to preoperative acute lung injury in 130 adults undergoing Stanford type-A acute aortic dissection surgery: a single-center cross-sectional clinical study. J Thorac Dis.

[37] Pellikka PA, She L, Holly TA, Lin G, Varadarajan P, Pai RG, Bonow RO, Pohost GM, Panza JA, Berman DS (2018). Variability in ejection fraction measured by echocardiography, gated single-photon emission computed tomography, and cardiac magnetic resonance in patients with coronary artery disease and left ventricular dysfunction. JAMA Netw Open.

[38] Nathan DP, Tang GL (2014). The impact of chronic renal insufficiency on vascular surgery patient outcomes. Semin Vasc Surg.

[39] Tian L, Fan X, Zhu J, Liang Y, Li J, Yang Y (2014). Plasma D-dimer and in-hospital mortality in patients with Stanford type A acute aortic dissection. Blood Coagul Fibrinolysis.

